# Development of a 5K Liquid-Phase Genome-Wide Breeding Chip for Xinglong Buffalo

**DOI:** 10.3390/ani15182702

**Published:** 2025-09-15

**Authors:** Yuqing Jiao, Junming Jiang, Shiyuan Li, Taoyu Chen, Xinjun Qiu, Ke Cui, Boling Li, Si Chen, Qiaoling Chen, Li Du, Churiga Man, Lianbin Li, Fengyang Wang, Hongyan Gao

**Affiliations:** 1 Hainan Key Laboratory for Tropical Animal Breeding and Disease Research, Hainan University, Haikou 570228, China; 324jyq@hainanu.edu.cn (Y.J.); jmm992847@163.com (J.J.); 22lishiyuan@hainanu.edu.cn (S.L.); chentaoyu@hainanu.edu.cn (T.C.); qiuxinjun@hainanu.edu.cn (X.Q.); chensi.ruth@hotmail.com (S.C.); chengiaoling1987@sina.cn (Q.C.); kych2008dl@163.com (L.D.); manchuriga@163.com (C.M.); lianbin@hainanu.edu.cn (L.L.); 2 School of Tropical Agriculture and Forestry, Hainan University, Haikou 570228, China; 3 The Hainan Animal Husbandry Technology Promotion Station, Haikou 571100, China; gzck020@163.com (K.C.); xmtgz4599@126.com (B.L.)

**Keywords:** Xinglong buffalo, single nucleotide polymorphism chip, genotyping by target sequencing, genetic evaluation

## Abstract

The Xinglong buffalo is a local swamp buffalo breed adapted to tropical regions in China. To facilitate the protection and utilization of valuable genetic resources, we first developed the breed-specific single nucleotide polymorphism (SNP) liquid-phase chip based on genotyping-by-target-sequencing (GBTS) technology. Whole-genome resequencing data from 143 buffaloes, resulting in 34,757,694 SNPs, were used to identify 1208 breed-specific and 2889 background sites. This chip also incorporates 965 functional SNP sites derived from literature, including SNPs significantly associated with immunity, reproduction, growth, and production. A total of 5062 SNP sites were successfully identified for the development of a 5K liquid-phase genome-wide breeding chip for the Xinglong buffalo. The validation of the chip using 93 samples showed a high detection rate with good repeatability and consistency. In addition, the chip exhibits strong capabilities in clustering and kinship analysis. Results of kinship analysis underscored the importance of a breed-specific chip for the Xinglong buffalo. These results highlight the advantages of a low-density, cost-effective, and breed-specific SNP chip for accurate genotyping. This chip will support future endeavors in molecular breeding, conservation, and genetic evaluation of Xinglong buffalo, thereby facilitating the sustainable utilization of this valuable indigenous germplasm resource.

## Simple Summary

The Xinglong buffalo is a local breed adapted to the tropical regions of Hainan Province, China, and possesses valuable genetic resources. With advancements in genomics and sequencing technologies, the development of single nucleotide polymorphism (SNP) chips has increased, enabling the evaluation, development, and utilization of local germplasm resources. In this study, we developed and evaluated a 5K liquid-phase SNP chip. The chip offers key advantages, including low cost and high specificity, while providing crucial genetic data to support future breeding programs. As such, it represents a strong foundation for the sustainable improvement of superior local breeds.

## 1. Introduction

Domestic buffalo are vital livestock in tropical and subtropical regions, providing milk, meat, and labor for many countries and regions [1]. There are two types of domestic water buffalo: swamp buffalo and river buffalo. They differ in chromosome number, phenotypic characteristics, and geographic distribution [1,2]. River buffalo are primarily raised for milk production and are mainly found in South Asia and Italy, while swamp buffalo are mainly raised for meat production and water-related labor, with a dominant presence in South China and Southeast Asia. The Xinglong buffalo, a superior breed in tropical regions of China, belongs to the swamp buffalo and is known for its exceptional tolerance to high humidity and heat, as well as its strong disease resistance. These traits have evolved through long-term adaptation to the tropical climate, genetic mutations, and selective breeding by local communities. Our previous research has indicated that the *FCRL5* gene, identified among the candidate genes of Xinglong buffalo, may play a significant role in innate immunity against tropical diseases [3]. Additionally, we identified certain genes (such as *GAMT*, *GCSH*, and *PNP*) and metabolites (such as L-aspartic acid, NADP+, and glutathione) associated with meat quality in Xinglong buffalo [4]. However, in recent years, their population has gradually declined, making it critical to implement effective measures to protect the germplasm resources.

Single nucleotide polymorphisms (SNPs) are widely used in animal genetic breeding research owing to their abundance, stable inheritance, broad distribution, and ease of detection [5]. SNPs are particularly valuable in applications such as genotyping chips [6], kinship identification [7], and marker-assisted selection [8]. SNP genotyping chips are commonly categorized into solid- and liquid-phase types. Compared with solid-phase chips, liquid-phase chip technology offers higher throughput, greater sensitivity, and multi-parameter analysis [9]. Genotyping by target sequencing (GBTS) technology enables the detection of target molecules binding to probes while determining their presence or absence, as well as their genotypes or specific variants [10]. Although whole-genome sequencing (WGS) provides detailed genomic information, including all genes and non-coding regions, its application also has some shortcomings. WGS is characterized by high costs, prolonged analysis duration, and complex analytical processes [11]. Compared to WGS, liquid-phase chips designed using GBTS technology have advantages in high detection efficiency, cost control, and target region coverage [12]. GBTS has been used to design liquid-phase chips for animals, including goats [13], sheep [14], cattle [15], and so on.

Existing buffalo SNP breeding chips, such as the Buffalo 50K SNP chip [16], the Axiom Buffalo Genotyping Array [17], and the Buffalo 90K SNP chip [18], are tailored for commercial breeds and do not recognize genetic markers unique to the Xinglong buffalo. As a result, these chips are less suitable for studies on the genetic diversity and evaluation of this local breed. To address this gap and facilitate the conservation and utilization of Xinglong buffalo germplasm resources, we applied GBTS technology to design a 5K liquid-phase SNP chip tailored to this breed. Its detection rate, repeatability, and grouping ability were validated, providing precise data to support future breeding and conservation of this unique breed.

## 2. Materials and Methods

All animal procedures were conducted in accordance with the Regulations for the Administration of Affairs Concerning Experimental Animals (Ministry of Science and Technology, China, 2004) and were approved by the owner’s informed consent and the Hainan University Institutional Animal Use and Care Committee (approval no. HNUAUCC-2025-00455; approval date: 26 May 2025).

### 2.1. Data Collection and SNP Calling

A total of 143 sequencing datasets were collected (Supplementary Table S1). The resequencing data of 15 Xinglong buffaloes were obtained from our previous study [3]. To examine the breed specificity of Xinglong buffaloes compared with other swamp buffalo breeds, we collected whole-genome resequencing data from 128 buffaloes representing 18 different breeds [19,20,21,22].

Raw sequencing reads, including low-quality reads and those with splices, were filtered using Fastp (version 0.20.0) with the following parameters: -n 10 -q 20 -u 40 [23]. After quality control, clean reads were aligned to the reference genome (*Bubalus carabanensis*/CUSA_SWP) of the swamp buffalo using the MEM algorithm in the BWA (version 2.2.1) [24]. SNPs were identified using the HaplotypeCaller module of GATK (version 4.0.4.0) [25]. Then, variants were filtered with the VariantFiltration module using the following parameters: --filter-expression “QD < 2.0 || QUAL < 30.0 || MQ < 40.0 || FS > 60.0 || SOR > 3.0 || MQRankSum < −12.5 || ReadPosRankSum < −8.0”.

### 2.2. Site Selection

Uniformly distributed SNPs with deletion rates below 30% and minor allele frequencies (MAFs) above 0.1 (indicative of high polymorphism) were selected as background loci.

Loci with deletion rates under 10% and heterozygosity rates below 50% were further screened. Samples and loci were divided into two groups: Xinglong and non-Xinglong buffaloes. ∆MAF values between the groups were calculated, and loci with ∆MAF values exceeding 0.2 were selected as specific sites.

Key traits, such as immunity, reproduction, growth, and production, were prioritized. Relevant loci were identified by reviewing functional information in literature available from PubMed (https://pubmed.ncbi.nlm.nih.gov/, accessed on 3 June 2024) and the China National Knowledge Infrastructure (CNKI; https://www.cnki.net/, accessed on 5 June 2024). To ascertain the precise locations of these SNP loci, the functional SNP genes identified were compared with the bovine reference genome.

### 2.3. Design and Synthesis of Probe

Selected sites were used for probe design and synthesis, adhering to the following criteria: each probe was designed to be 110 bp in length with a GC content of 30–70%, each SNP site was completely covered by the probe, homologous regions (exhibiting high sequence similarity) were limited to fewer than five, and all SNP sites were evenly distributed. After evaluating the probe design, the probes were synthesized and tested. The designed probe was removed for sites with poor capture rates. Only sites meeting all criteria were included to develop the SNP liquid-phase chip for the Xinglong buffalo. The design strategy and verification experiments of a 5K liquid-phase chip for Xinglong buffalo are shown in Supplementary Figure S1.

### 2.4. Functional Analysis of Liquid-Phase Chip Loci

The SNP loci were annotated using ANNOVAR (version 7 June 2020) [26] with the buffalo reference genome. Loci located in exonic regions were selected. Gene Ontology (GO) and Kyoto Encyclopedia of Genes and Genomes (KEGG) analyses were performed using the Database for Annotation, Visualization and Integrated Discovery (DAVID, https://davidbioinformatics.nih.gov/, accessed on 14 October 2024) [27].

### 2.5. DNA Extraction and Sequencing Library Construction

Ear tissues of Xinglong buffalo were obtained from local farms in Ding’an County, Hainan Province, China. The experimental Xinglong buffaloes were not euthanized or slaughtered. Xinglong buffaloes were restrained in a squeeze chute in the presence of a veterinarian. After shaving and disinfecting the ear, surface anesthesia was administered using 2% lidocaine hydrochloride [28]. Afterwards, a 2–3 mm tissue sample was collected from the outer edge of the ear using an ear punch. DNA was extracted from the ear tissue samples of 98 Xinglong buffaloes using the TIANamp Genomic DNA Kit (Tiangen, Beijing, China). The purity and integrity of the extracted DNA were assessed using 1% agarose gel electrophoresis, and DNA concentrations were accurately quantified using Qubit 2.0 (Invitrogen, Shanghai, China). A multiplex polymerase chain reaction (PCR) was conducted by adding a PCR panel mix and multiplex PCR amplification enzyme system to DNA that passed quality control. The resulting PCR products were purified and underwent further amplification. After additional purification using carboxyl beads, multiplex PCR capture and library construction were completed. After library construction, preliminary quantification was performed using Qubit 2.0, and the effective library concentration was accurately determined by quantitative PCR. Once qualified, libraries were sequenced.

### 2.6. Verification of Liquid-Phase Chip

To validate the ability of the Xinglong buffalo 5K liquid-phase chip to detect SNP site information, the SNP detection rate was tested using DNA samples from 98 individuals. Five samples with insufficient downstream data were excluded from the analysis. Key metrics, including total loci count, locus deletion rate, heterozygosity rate, and minor allele frequency, were obtained through bioinformatics analysis.

Additionally, to assess the accuracy of chip genotyping, we compared SNP genotyping results with resequencing data [29]. Fifteen Xinglong buffalo samples previously analyzed by whole-genome resequencing were tested on the chip, and their SNP results were compared with those obtained through resequencing. Four DNA samples among the ninety-eight samples were randomly selected for duplicate testing, and repeatability was determined by comparing results from replicate samples.

### 2.7. Validation of Non-Synonymous Mutation Sites

Non-synonymous mutation sites were selected from the screened chip-specific loci, and primers were designed for approximately 300 bp upstream and downstream of the mutation sites. The total volume of the PCR reaction was 30 µL, which included 1 µL DNA, 1 µL of each upstream and downstream primers, 7 µL H_2_O, and 20 µL 2× Accurate Taq Mix (dye) (Aikerui, Changsha, Hunan, China). The PCR program was predenatured for the first 4 min, followed by 31 cycles of denaturation at 94 °C for 30 s, annealing at 59 °C for 30 s, extension at 72 °C for 27 s, and a final extension at 72 °C for 10 min. The PCR products were analyzed by 1.5% agarose gel electrophoresis to assess their quality. Subsequently, the PCR products were sequenced and analyzed.

### 2.8. Analysis of Breed and Kinship

To assess the chip’s capability in detecting population structure, its ability to distinguish between different pedigrees of Xinglong buffalo was evaluated. Genotyping results from 93 Xinglong buffalo DNA samples were combined with resequencing data from 60 non-Xinglong buffalo samples to produce a VCF file using BCFtools (version 1.20) [30]. Maximum likelihood trees were constructed using IQ-TREE (version 2.3.6) [31] with a bootstrap value of 1000 generations. Principal component analysis (PCA) [32] was performed using PLINK (version 1.9) [33] and visualized with the R-4.3.1 package for PCA landscaping [34]. The phylogenetic tree landscape was generated using an online tool [35]. Additionally, kinship analysis of the genotyping results from 93 Xinglong buffalo DNA samples was conducted using TASSEL (version 5.2.94) [36].

## 3. Results

### 3.1. Identification of Functional and Specific Sites

After removing duplicates and incomplete loci, information on SNPs from 68 publications in PubMed and CNKI was recorded (Supplementary Table S2), and 1039 candidate SNP loci linked to important traits were selected.

**Figure 1:**
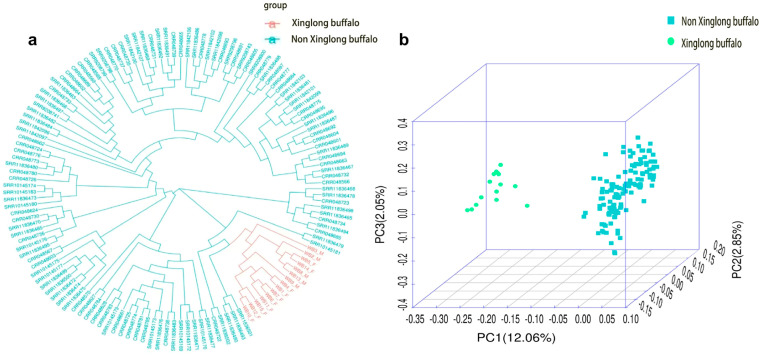
Phylogenetic tree and PCA results for 143 buffalo samples. (**a**) Phylogenetic tree; (**b**) PCA results.

A total of 143 resequencing datasets were analyzed. After quality control, the effective rate of reads was between 93.99% and 99.98%, with an average effective rate of 99.17%. Then, the clean reads were aligned to the reference genome with an average alignment rate of 94.84%. A total of 34,757,694 SNPs were subsequently identified across all samples. The phylogenetic tree analysis and PCA were performed on the filtered data (Figure 1). The phylogenetic tree revealed that Xinglong buffalo diverged from other breeds, while PCA revealed that PC1 and PC2 accounted for 12.06% and 2.85% of the variance, respectively. These findings confirmed the successful construction of Xinglong and non-Xinglong buffalo populations. By calculating the ∆MAF values between Xinglong and non-Xinglong buffaloes, 60,488 loci were identified as breed-specific loci. Additionally, 5,299,710 loci were selected as background loci.

### 3.2. Analysis of SNP Loci on the 5K Liquid-Phase Chip

**Figure 2:**
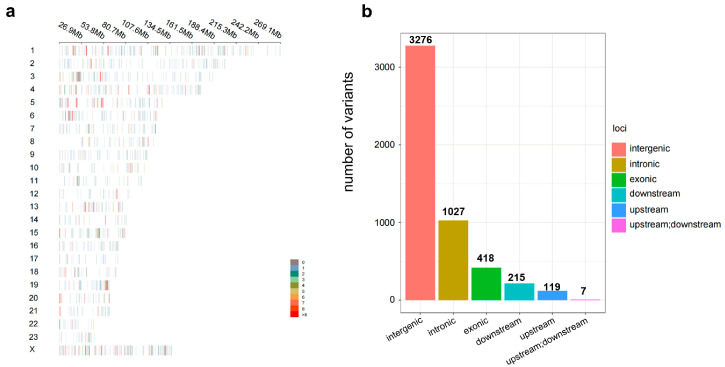
Analysis of SNP loci on the 5K liquid-phase chip. (**a**) Chromosomal distribution of SNP loci. (**b**) Annotation results of core loci on the 5K liquid-phase chip. Upstream: SNP loci located within 2 Kbp upstream of the gene; downstream: SNP loci located within 2 Kbp downstream of the gene; upstream;downstream: variant located in both downstream and upstream regions (possibly for two different genes).

Probes were designed for the identified loci, including 1039 functional loci from the literature, according to established screening principles. In total, 5871 loci were selected, comprising 965 functional, 1208 breed-specific, and 3698 background sites. These loci were synthesized and tested, with 5062 loci (a total of 9793 probes) retained for chip design and development. The uniform distribution of 5062 SNP sites was observed (Figure 2a). Annotation of these loci revealed that most SNPs (85.01%) were located either between genes or within introns. Additionally, 8.26% of SNPs were located within exon coding regions (Figure 2b).

### 3.3. Functional Analysis of 5K Liquid-Phase Chip

**Figure 3:**
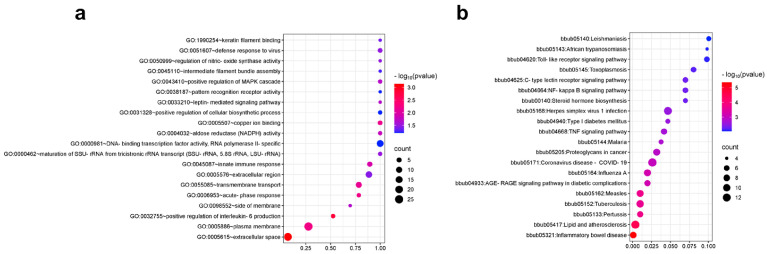
GO and KEGG analyses of genes with SNP annotations in exon regions. (**a**,**b**) Scatter plots of the top 20 significantly enriched (**a**) GO terms and (**b**) KEGG pathways.

A total of 221 genes were annotated from chip loci located in exonic regions. GO enrichment analysis revealed thirteen biological processes, six molecular functions, and six cellular components. The top 20 significantly enriched terms across these categories are visualized (Figure 3a). Notable immune-related GO terms were as follows: extracellular space (GO:0005615), positive regulation of interleukin-6 production (GO:0032755), acute-phase response (GO:0006953), innate immune response (GO:0045087), defense response to virus (GO:0051607), and extracellular region (GO:0005576). KEGG analysis identified 44 significantly enriched pathways, with the top 20 visualized (Figure 3b). Immune-related pathways included the following: inflammatory bowel disease (bbub05321), TNF signaling pathway (bbub04668), NF-kappa B signaling pathway (bbub04064), Toll-like receptor signaling pathway (bbub04620), and C-type lectin receptor signaling pathway (bbub04625). In addition to those listed above, most of the other terms are also indirectly related to immunity. These results suggest that the 5K liquid-phase chip can effectively identify SNPs linked to immune traits in Xinglong buffalo.

### 3.4. Verification of 5K Liquid-Phase Chip

**Figure 4:**
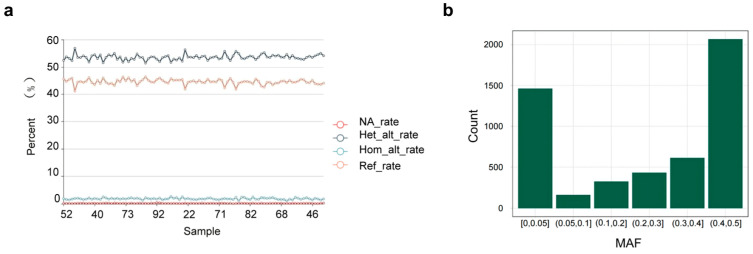
Chip validation results. (**a**) Statistical chart showing all Xinglong buffaloes (horizontal coordinate, sample names; vertical coordinate, percentages); (**b**) MAF distribution of SNP loci detected in all Xinglong buffalo samples using the 5K liquid-phase chip.

The detection rates for target SNP loci in validation samples were 99.57–99.96%, with an average detection rate of 99.82%. Figure 4a shows site deletions and mutations for each sample, and Figure 4b illustrates the MAF site distribution, highlighting the highest and lowest SNP frequencies: 0.4–0.5 and 0.05–0.10, respectively.

### 3.5. Consistency and Repeatability Analysis of the Chip

The consistency between genotyping results and resequencing results ranged from 85.62% to 89.66%, with an average consistency of 87.73%. Repeatability was further evaluated using four samples (buffalo-5, -6, -12, and -14) by comparing duplicate genotyping results, yielding a concordance rate of 99.90–99.96% (Table 1).

### 3.6. Validation of Non-Synonymous Mutation Sites of Liquid-Phase Chip

Eight non-synonymous mutation sites were identified from the specific loci of the chip, and the genes annotated by these loci include *LOC102398488*, *LOC123329764*, *LOC102414696*, *LOC102401256*, *LOC102392256*, *FBXO16*, *LOC123465813*, and *PLCXD1*. In particular, PLCXD1 is a member of the phosphatidylinositol-specific phospholipase C family. Research has shown that PLCXD1 regulates various cellular processes by modulating cytosolic calcium levels and/or influencing the activity of several protein kinases [37]. FBXO16 is classified as a member of the F-box protein family and is a component of the SCF (SKP1-Cullin1-F-box) ubiquitin ligase complex, playing a crucial role in protein ubiquitination and degradation [38,39,40]. Verification of two loci revealed that they were indeed non-synonymous mutation loci (Table 2, Supplementary Figure S2).

### 3.7. Analysis of Breed and Kinship Based on the Chip Data

**Figure 5:**
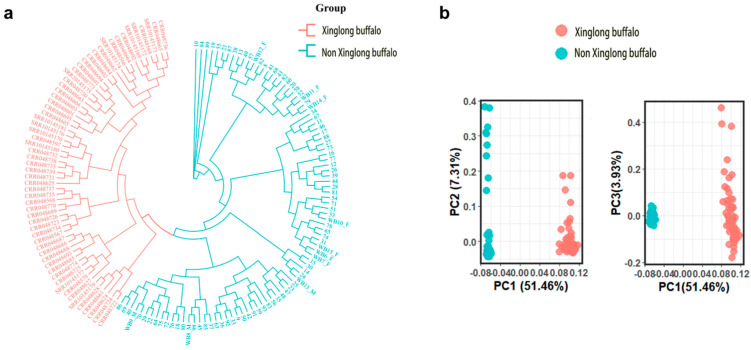
Phylogenetic tree and PCA results based on the 5K liquid-phase chip analysis of the Xinglong buffalo. (**a**) Phylogenetic tree; (**b**) PCA results.

**Figure 6:**
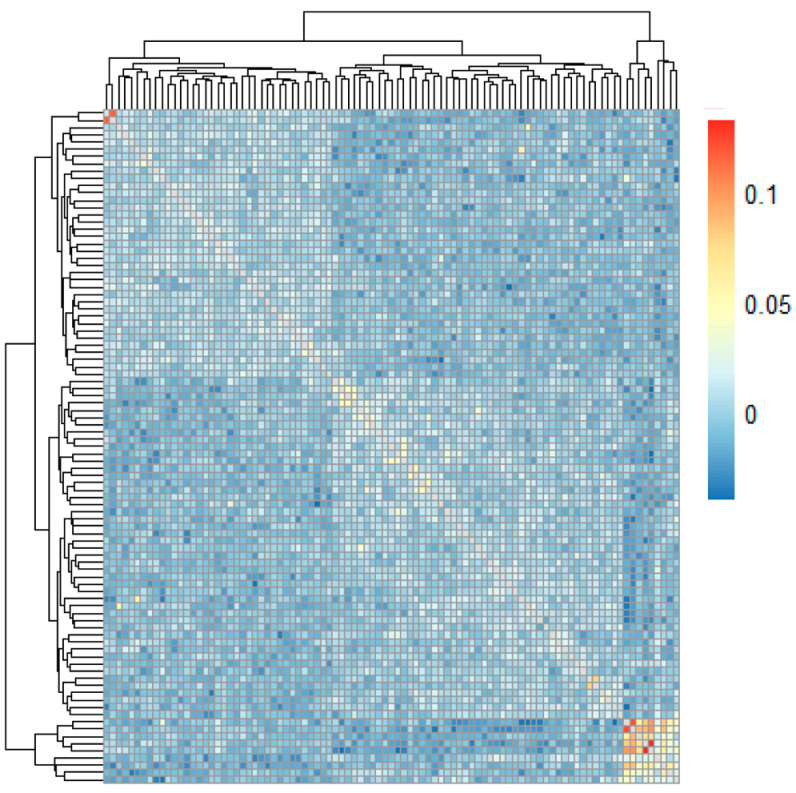
Kinship analysis of 93 Xinglong buffalo samples.

Cluster analysis was conducted using genotyping data from 153 samples to evaluate subgrouping capabilities. Phylogenetic tree construction and PCA revealed distinct clustering of the Xinglong buffalo from others (Figure 5). Kinship analysis of the 93 Xinglong buffalo samples identified three clusters (Figure 6), with closely related individuals in each cluster (indicated by darker squares), highlighting the likely inbreeding within these groups.

## 4. Discussion

Currently, the buffalo breeding chip is tailored for commercial breeds and does not contain specific SNP information related to Xinglong buffalo. Therefore, to support the development and conservation of this valuable genetic resource in the tropical regions of China, we have developed a 5K SNP liquid-phase chip specifically for Xinglong buffalo.

The SNP loci included in the 5K liquid-phase chip of the Xinglong buffalo were derived from two primary sources: literature and whole-genome resequencing data. Relevant SNPs were identified by searching for genes associated with critical cattle traits, including immunity, reproduction, growth, and production. This approach led to the selection of 965 functional loci, which are associated with traits that considerably impact economic performance and the overall health of buffaloes. A similar strategy was used in the development of the Populus trichocarpa 34K SNP genotyping array, where candidate genes were identified through extensive literature and database searches [41]. Likewise, during the development of the chicken 55K SNP genotyping array, SNPs associated with economically important traits were prioritized for inclusion [42].

Based on the analysis of whole-genome resequencing data from 143 buffaloes, we identified 2889 background loci and 1208 specific loci. Using the ∆MAF value allows for the screening of unique SNP sites in the Xinglong buffalo, facilitating better identification of the breed. The variability in MAF across populations highlights its utility as a marker for genetic differentiation [43,44,45]. Prior studies have underscored the robustness and relevance of absolute allele frequency differences as indicators of genetic divergence [46,47]. When screening for background loci, selecting SNPs with high-frequency distributions is critical, as low-frequency loci often provide insufficient variation for downstream analyses. During the validation of the Xinglong buffalo liquid-phase chip, although most SNP loci exhibited MAFs greater than 0.01, 950 SNPs showed MAF values below 0.01. This may reflect the exclusive use of Xinglong buffaloes during validation, potentially excluding variants with low allele frequencies present in other populations. Low-frequency SNPs typically provide limited information [48], emphasizing the importance of using diverse populations and larger sample sizes in future chip validation efforts.

Annotation of the chip loci revealed that the majority of SNPs were located in intergenic or intronic regions, collectively accounting for approximately 85.06% of the total core SNP loci. Although these regions do not directly encode proteins, they are crucial for regulating gene expression and other biological functions. For instance, Guo et al. [49] reported that 74.3% of marker SNPs in their 40K maize chip were intergenic, 15.3% were intronic, and only 6.2% originated from other regions. Similarly, Stothard et al. [50] analyzed SNPs derived from whole-genome resequencing of Black Angus and Holstein cattle, finding that most SNPs in Holstein cattle were intergenic (66.3%) or intronic (26.7%), with similar distributions in Black Angus cattle (intergenic, 65.7%; intronic, 27.2%). By correlating SNP loci with phenotypic traits, our findings contribute to future studies on gene localization and molecular marker-assisted breeding in buffalo populations.

To investigate the functional significance of the SNP loci, we performed GO and KEGG enrichment analyses of 221 genes associated with exonic SNPs identified in the 5K liquid-phase chip for the Xinglong buffalo. The top 20 enriched GO terms and KEGG pathways primarily involved genes associated with immune responses and disease susceptibility. The extracellular space (GO:0005615) is the first term enriched in GO terminology. Gaggero et al. [51] elucidated the direct regulatory role of the extracellular space microenvironment on immune signaling by investigating the signaling mechanism of cytokines in the extracellular space. Inflammatory bowel disease (bbub05321) ranks as the foremost enrichment in the KEGG pathway. Esposito et al. [52] stated that inflammatory bowel disease is an immune-mediated disease resulting from dysregulation of the intestinal immune response. These findings suggest that the 5K chip successfully captures SNPs linked to immune- and disease-related pathways. Considering the disease resistance and adaptability of the Xinglong buffalo, these immune-related genes (such as *NOD2*, *BCL2*, *TLR4,* and *HSP70*) provide mechanistic support for their adaptive traits. Specifically, SNP mutations in *NOD2* influence mammary inflammatory responses, and variants in *BCL2* are associated with both mammary inflammation and heat stress tolerance [53]. SNP mutations in *TLR4* influence the recognition of bacterial infections [54]. The mutations in *HSP70* affect the cellular defense mechanism under heat stress conditions [55]. Collectively, these results indicate that the chip offers a valuable resource for breeding programs aimed at enhancing disease resistance and adaptability in Xinglong buffalo.

The detection rates for target SNP loci in validation samples were 99.57–99.96%, with an average detection rate of 99.82%. This performance is comparable to or even exceeds that of other livestock breeding chips [14,29,56]. Concordance between chip genotyping and resequencing results ranged from 85.62% to 89.66%. Variability in sequencing depth likely contributed to this moderate concordance. Wei et al. [57] reported that the average concordance rate of SNP genotyping between their SNP array and resequencing data was 84.07%. To further evaluate accuracy, they analyzed 18 inconsistent SNPs using Sanger sequencing and found that neither resequencing nor SNP arrays achieved 100% accuracy. In this study, further repeatability tests using duplicate samples from four individuals showed consistency rates ranging from 99.90% to 99.96%. These results demonstrate the chip’s exceptional repeatability and confirm that it meets the requirements for genotyping applications in Xinglong buffaloes.

In addition, two non-synonymous loci were selected from the specific loci of the chip for validation. The genes associated with these loci are *PLCXD1* and *FBXO16*. The *PLCXD1* gene plays a crucial role in regulating intracellular calcium and inositol phosphate balance [58]. Intracellular calcium ions (Ca^2+^) act as key secondary messengers, closely linked to various physiological and pathological processes [59,60,61]. Phosphoinositides are essential membrane lipids that regulate numerous dynamic cellular processes, from cell migration to mitosis [62]. The *FBXO16* gene functions as a tumor suppressor [40], and recent studies have shown that the *FBXO16* gene mediates the degradation of NF-κB p65 subunits, thereby inhibiting inflammatory responses in dendritic cells [63]. By verifying these two specific sites, it was confirmed that they were indeed non-synonymous mutations. The results were consistent with the 93 chip detection results, further validating the accuracy of this chip.

Phylogenetic tree and PCA results showed distinct clustering of Xinglong buffalo populations, validating the chip’s ability to differentiate populations. Samuel et al. [64] identified three SNPs for the bovine prolactin gene, and one of the mutations affects milk yield. Therefore, the genotyping results enable us to ascertain the mutation status of the locus gene in each sample, facilitating the selection for breeding high-quality Xinglong buffaloes. Additionally, genotyping results can be used for kinship analysis. Kinship analysis, which is vital for animal breeding and conservation [65,66], revealed that most of the 93 Xinglong buffaloes analyzed were closely related, with evidence of possible inbreeding. This finding underscores the need for genetic management strategies to conserve and improve the valuable germplasm of Xinglong buffaloes. Developing specialized SNP liquid-phase chips for this breed will be critical for its sustainable conservation and genetic improvement.

Currently, genotyping costs remain relatively high. Existing buffalo SNP breeding chips are tailored for commercial breeds and do not recognize genetic markers unique to the Xinglong buffalo. Therefore, the 5K SNP liquid-phase genome-wide breeding chip was specifically developed for the Xinglong buffalo in this study. Compared to whole-genome resequencing and high-density solid-phase chips, this chip not only significantly reduces detection costs but, more importantly, its SNP loci have been meticulously selected to fully align with the genetic characteristics and breeding requirements of the Xinglong buffalo. This customized liquid-phase chip technology offers efficient and accurate screening capabilities, enabling precise identification of superior individuals with desirable traits, such as growth performance and disease resistance, which are particularly important for the Xinglong buffalo. The application of this chip will provide crucial technical support for the conservation and selective breeding of this valuable local breed, significantly accelerating the breeding progress. In addition, liquid-phase chips have high flexibility and extensibility; SNP loci significantly associated with functional genes can be added to the chip system as needed in subsequent research without modifying the existing loci [10,67]. This not only optimizes chip loci for the genetic background of Xinglong buffalo but also enhances the real-time updating of selection-relevant loci in its genome.

## 5. Conclusions

We successfully developed a 5K liquid-phase chip for the Xinglong buffalo by screening 5062 SNP loci, including functional SNPs associated with key traits, the association of which needs to be confirmed in further studies for the Xinglong buffalo. Validation results confirmed that the chip meets the performance requirements for Xinglong buffalo genotyping. This cost-effective genotyping tool is particularly suited for breed identification, kinship analysis, and germplasm conservation. Moreover, the chip provides a scientific basis for crossbreeding and genetic improvement while aiding in the conservation and development of Xinglong buffalo resources. By enabling accurate genotyping, the chip enhances the ability to identify valuable genetic traits, thereby supporting buffalo breeding initiatives.

## Figures and Tables

**Table 1:** Genotypic concordance rates for duplicate samples.

Sample ID	Number of Discordant SNPs	Concordance Rate
buffalo-5 and buffalo-5-re	5	99.90%
buffalo-6 and buffalo-6-re	3	99.94%
buffalo-12 and buffalo-12-re	3	99.94%
buffalo-14 and buffalo-14-re	2	99.96%

**Table 2:** The SNP information of genes (*PLCXD1* and *FBXO16*).

Gene	Location	SNP *	Attribute	Amino Acid
*PLCXD1*	chrX:136183705	c.464C>T	non-synonymous	p.155A>V
*FBXO16*	chr4:71852182	c.421T>G	non-synonymous	p.141F>V

* c. represents the coding sequence region.

## Data Availability

The genomic sequences of 15 Xinglong buffaloes have been deposited in the Genome Sequence Archive (GSA) under accession number CRA014333 (https://ngdc.cncb.ac.cn/gsa/browse/CRA014333, accessed on 18 December 2024), and information on the genomic sequences of 128 buffaloes is provided in Supplementary Table S1.

## Supplementary Materials

The following supporting information can be downloaded at: https://www.mdpi.com/article/10.3390/ani15182702/s1, Table S1: Source of 143 buffaloes Genome Sequences; Table S2: Information collected from the article; Figure S1. The design strategy and verification experiments of a 5K liquid-phase chip for Xinglong buffalo; Figure S2. The results of sequencing. (a) the results of PLCXD1; (b) the results of FBXO16.

## Abbreviations

SNP	Single nucleotide polymorphism
GBTS	Genotyping by target sequencing
MAF	Minor allele frequency
GO	Gene ontology
KEGG	Kyoto Encyclopedia of Genes and Genomes
PCA	Principal component analysis
PCR	Polymerase chain reaction
