# Supplementary material for: Development of a 5K Liquid-Phase Genome-Wide Breeding Chip for Xinglong Buffalo
Source: Animals (Basel). 2025 Sep 15;15(18):2702. doi: 10.3390/ani15182702 (PMC12466515; doi:10.3390/ani15182702)
Supplement: Supplementary file 1 [file animals-15-02702-s001.zip › Supplementary Figures S1 and S2.pdf]

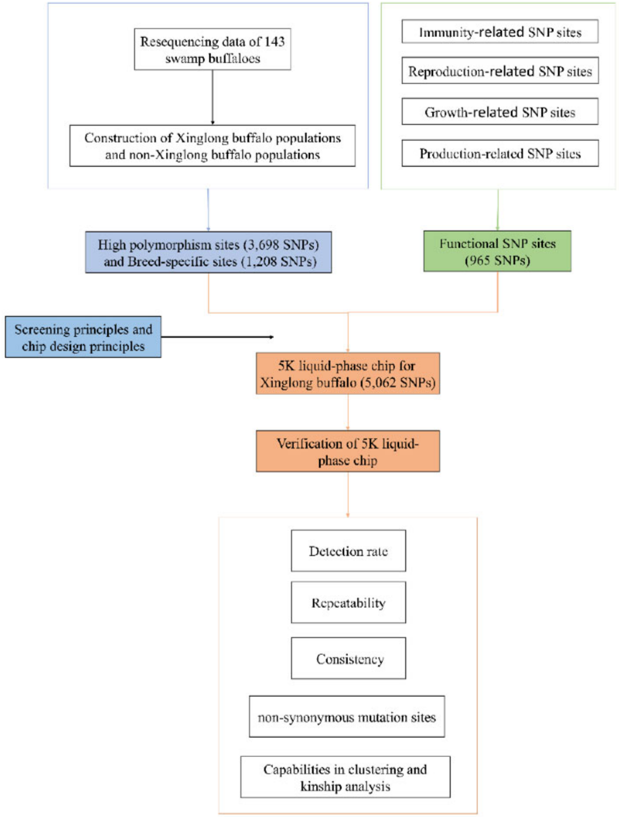

Supplementary Figure S1. The design strategy and verification experiments of a 5K liquid-phase chip for Xinglong buffalo.

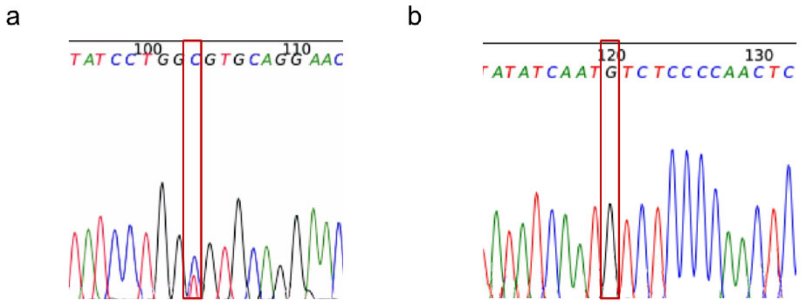

Supplementary Figure S2. The results of sequencing.(a) the results of PLCXD1; (b) the results of FBXO16.
